# Acupuncture combined with pelvic floor rehabilitation training for postpartum stress urinary incontinence: protocol for a systematic review and meta-analysis

**DOI:** 10.3389/fmed.2023.1296751

**Published:** 2023-12-06

**Authors:** Chu Wenming, Deng Xiaoman, Gao Ling, Li Yun, Gao Xiyan

**Affiliations:** ^1^College of Acupuncture and Tuina, Henan University of Chinese Medicine, Zhengzhou, Henan Province, China; ^2^Department of Acupuncture and Rehabilitation, Affiliated Hospital of Nanjing University of Chinese Medicine, Nanjing, Jiangsu Province, China; ^3^Department of Acupuncture, The Third Affiliated Hospital of Henan University of Chinese Medicine, Zhengzhou, Henan Province, China

**Keywords:** acupuncture, pelvic floor rehabilitation training, integrative traditional Chinese and western medicine, postpartum, stress urinary incontinence

## Abstract

**Introduction:**

The purpose of this study is to systematically evaluate the efficacy and safety of acupuncture combined with pelvic floor rehabilitation training in the treatment of postpartum stress urinary incontinence, and to promote the further promotion and application of acupuncture in the field of rehabilitation.

**Methods and analysis:**

Randomized controlled trials (RCTs) of acupuncture combined with pelvic floor rehabilitation in the treatment of postpartum stress urinary incontinence will be searched in PubMed, Web of Science (WOS), Cochrane Library, EMBASE, China National Knowledge Infrastructure (CNKI), Chinese Biomedical Literature Database (CBM), Wanfang (WF), and VIP databases. The clinical trial Registry (ClinicalTrials.gov and Chinese clinical trial Registry) will also be searched. The search period is limited to July 1, 2023, and the language limit of this systematic review is Chinese and English. The primary outcome is clinical effective rate. International Consultation on Incontinence Questionnaire-Short Form (ICI-Q-SF), 1-h pad test, pelvic floor muscle potential value, incidence of adverse events are secondary outcomes. A meta-analysis will be performed using RevMan 5.4 statistical software. If feasible, subgroup analysis and sensitivity analysis will be performed to address potential causes of inconsistency and heterogeneity. The risk of bias will be assessed using the approach recommended by *Cochrane Handbook for Systematic Reviews of Interventions*, and the quality of evidence will be assessed using GRADE. This Protocol has been developed in accordance with the guideline of *Preferred Reporting Items for Systematic review and Meta-Analysis Protocols (PRISMA-P) 2015*.

**Discussion:**

Acupuncture combined with pelvic floor rehabilitation training can effectively promote the rehabilitation of postpartum stress urinary incontinence patients, and provide a reference for the clinical application of integrated Chinese and Western medicine treatment in the field of rehabilitation.

**Systematic review registration:**

PROSPERO CRD42023455801.

## 1 Introduction

Postpartum Stress Urinary Incontinence (PSUI) refers to the occurrence of urinary incontinence after pregnancy or childbirth. The main symptoms are urine leakage from the urethra when the maternal abdominal pressure increases, such as coughing, sneezing, laughing, walking or running. Symptoms stopped immediately after stopping these actions (1). The causes of PSUI are mainly related to the hyperextension or injury of pelvic floor muscles, surrounding connective tissues and pelvic floor nerves during vaginal delivery. Risk factors associated with PSUI included higher maternal body mass index (BMI), age, delivery time, vaginal delivery, surgical vaginal delivery, perineal or anal sphincter trauma, and higher birth weight (2–6). According to relevant studies, the incidence of stress urinary incontinence is about 19% after 6 weeks (7). In a systematic review of population-based studies, 33% of women experienced urinary incontinence within the first 3 months after giving birth (8). A longitudinal cohort study showed that the prevalence of stress incontinence reached 42% in 241 women 12 years after their first birth. The prevalence of stress incontinence during the first pregnancy and 12 years after delivery was significantly higher in women who developed it during the first pregnancy (56%) and shortly after delivery (78%) than in women without initial symptoms (30%), indicating an increased risk of long-term symptoms of stress incontinence occurring during the first pregnancy or puerperal period (9). This disease not only seriously affects the quality of life of patients, but also increases the psychological burden and economic burden of patients (10, 11). In order to allow the body to recover from pregnancy and childbirth, surgical treatment is usually delayed after delivery. Medication may not be appropriate for breastfeeding women (12). Stem cell research, which has risen to the forefront of regenerative medicine, has the potential to cure stress urinary incontinence but has not yet been approved for routine clinical usage (13).

Pelvic floor muscle-related symptoms may coexist with symptoms of pelvic floor disorders such as urinary incontinence, voiding dysfunction, fecal incontinence, defecatory dysfunction, sexual dysfunction, or pelvic organ prolapse, as well as coexist with other disorders of neuro-musculo-skeletal structures in the pelvis or spine (14). Studies recommend pelvic floor rehabilitation training as the first choice for conservative treatment of stress incontinence (15). The Chinese *Guidelines for the Diagnosis and Treatment of female stress Urinary Incontinence* (2017) recommend pelvic floor muscle training as a first-line non-surgical treatment, with efficacy somewhat similar to surgery and without any risk (1). Pelvic floor rehabilitation training can effectively alleviate postpartum pelvic floor dysfunction and reduce symptoms of urinary incontinence. New study found that pelvic floor rehabilitation training protocol reduced urinary incontinence in pregnant women. The program allowed significant improvement in the quantity of urinary leakage and an increase in the strength of the pelvic floor muscle (16). Conventional pelvic floor rehabilitation methods mainly include Kegel pelvic floor muscle training, Biofeedback training, low-frequency bioelectrical stimulation, and the use of vaginal dumbbell (17). However, the poor compliance of patient’s leads to poor recovery effect is a common phenomenon in society. It is often due to factors such as inability to adhere to a family exercise plan, inconvenience in daily activities, worry about children, uncertainty about efficacy, and financial burden (18). Therefore, the effect of single treatment is limited (19).

Acupuncture, as an important traditional Chinese medicine treatment, has been included in the Representative List of the Intangible Cultural Heritage of Humanity by the United Nations Educational, Scientific and Cultural Organization (UNESCO) and is considered safe and effective (20–22). Acupuncture is applicable to a wide range of diseases. In a systematic study that identified 133 acupuncture-related clinical practice guidelines and 433 acupuncture interventions published between 2010 and 2019, 380 recommended the use of acupuncture, and 43.2% of the 303 recommendations that used GRADE to determine the strength of recommendations were strong. Acupuncture is recommended in 87.8% of obstetrics, gynecology and women’s guidelines (23). Acupuncture has certain advantages in the treatment of PSUI, which can regulate the pelvic floor muscle through the stimulation of relevant acupuncture points, thus promoting the recovery of bladder function (24). In recent years, the integration of Chinese and Western medicine in treating various diseases has gained significant attention in research. Acupuncture combined with pelvic floor rehabilitation training has made certain progress in improving the clinical symptoms of PSUI patients (25–28), but there is still a lack of high-quality systematic reviews at home and abroad. Only one systematic review evaluated the effectiveness of acupuncture combined with pelvic floor muscle training for PSUI (29). The review has obvious flaws that threaten the veracity of their findings. Firstly, this study was not registered in the *International Prospective Register of Systematic Reviews (PROSPERO)*. Secondly, it is found that the search scope was small, only 6 databases were searched. Thirdly, the methodological quality of the 12 included papers was very poor. Finally, acupuncture is treated as a whole, and the efficacy of acupuncture alone has not been systematically studied. From what we know, several new RCTs have been published since the meta-analysis was published.

In conclusion, there is no supporting evidence for acupuncture combined with pelvic floor rehabilitation training to improve the efficacy and safety of PSUI. This study collected domestic and foreign relevant clinical research literature for quality evaluation and meta-analysis, in order to provide scientific evidence for acupuncture combined with pelvic floor rehabilitation training to improve PSUI in clinical practice.

## 2 Methods and analysis

We constructed this protocol report and implementation review according to the guidelines for the *Preferred Reporting Items for Systematic review and Meta-Analysis Protocols (PRISMA-P) 2015* (30).

### 2.1 Study registration

In accordance with the guidelines, our systematic review protocol was registered with *PROSPERO* on August 30, 2023 (registration number CRD42023455801).

### 2.2 Eligibility criteria

#### 2.2.1 Study designs

We will only include randomized controlled trials (RCTs), excluding cross-sectional studies, reviews, case reports and systematic reviews.

#### 2.2.2 Participants

We will include women who meet any of the diagnostic criteria or guidelines for postpartum stress incontinence and exclude patients with serious other psychiatric disorders. There will be no restrictions on the age, race, education level, course of disease, etc.

#### 2.2.3 Interventions

The experimental group will include any kind of acupuncture therapy combined with pelvic floor rehabilitation training, including acupuncture, moxibustion, electroacupuncture, warm acupuncture, etc. Non-acupuncture combined with pelvic floor rehabilitation training intervention, such as acupuncture combined with pelvic floor rehabilitation training combined with massage, Chinese medicine, nursing and other confounding factors will be excluded.

#### 2.2.4 Comparators

The control group will only be included in the simple pelvic floor rehabilitation training, including Kegel pelvic floor muscle training, Biofeedback training, low-frequency bioelectrical stimulation, vaginal dumbbell (17, 31).

#### 2.2.5 Outcomes

*Primary outcome*: The primary outcome of this systematic review will be clinical effective rate.

*Secondary outcomes*: International Consultation on Incontinence Questionnaire-Short Form (ICI-Q-SF) (32), 1-h pad test, pelvic floor muscle potential value, incidence of adverse events will be included as secondary outcomes.

#### 2.2.6 Language

We will only include articles reported in Chinese and English.

### 2.3 Search strategy

Randomized controlled trials (RCTs) of acupuncture combined with pelvic floor rehabilitation in the treatment of postpartum stress urinary incontinence will be searched in PubMed, Web of Science (WOS), Cochrane Library, EMBASE, China National Knowledge Infrastructure (CNKI), Chinese Biomedical Literature Database (CBM), Wanfang (WF), and VIP databases. The clinical trial Registry (ClinicalTrials.gov and Chinese clinical trial Registry) will also be searched. The search period will be limited to July 1, 2023, and the language limit of this systematic review will be Chinese and English. Subject words combined with free words will be used in the search. The search strategy for PubMed is shown in Table 1.

**TABLE 1:** Search strategy for PubMed.

Search	Query
#1	“Urinary Incontinence, Stress”[MeSH Terms] OR “stress urinary incontinence”[Title/Abstract] OR “urinary incontinence”[Title/Abstract] OR “enuresis”[Title/Abstract]
#2	“Postpartum Period”[MeSH Terms] OR “period postpartum”[Title/Abstract] OR “Postpartum”[Title/Abstract] OR “Postpartum Women”[Title/Abstract] OR “women postpartum”[Title/Abstract] OR “Puerperium”[Title/Abstract]
#3	“Acupuncture”[MeSH Terms] OR “Pharmacopuncture”[Title/Abstract] OR “Acupuncture therapy”[Title/Abstract] OR “Acupuncture Treatment”[Title/Abstract] OR “Moxibustion”[Title/Abstract] OR “Electroacupuncture”[Title/Abstract] OR “Warm Acupuncture”[Title/Abstract] OR “Needling”[Title/Abstract] OR “Body Acupuncture”[Title/Abstract] OR “Acupotomy”[Title/Abstract] OR “Abdominal Acupuncture”[Title/Abstract] OR “Fire Needling”[Title/Abstract]
#4	“Rehabilitation”[MeSH Terms] OR “pelvic floor rehabilitation”[Title/Abstract] OR “pelvic floor muscle training”[Title/Abstract] OR “PFMT”[Title/Abstract] OR “Kegel movement” [Title/Abstract] OR “Biofeedback training”[Title/Abstract] OR “low-frequency bioelectrical stimulation”[Title/Abstract] OR “vaginal dumbbell”[Title/Abstract]
#5	#1 AND #2 AND #3 AND #4

### 2.4 Selection of studies

**FIGURE 1:**
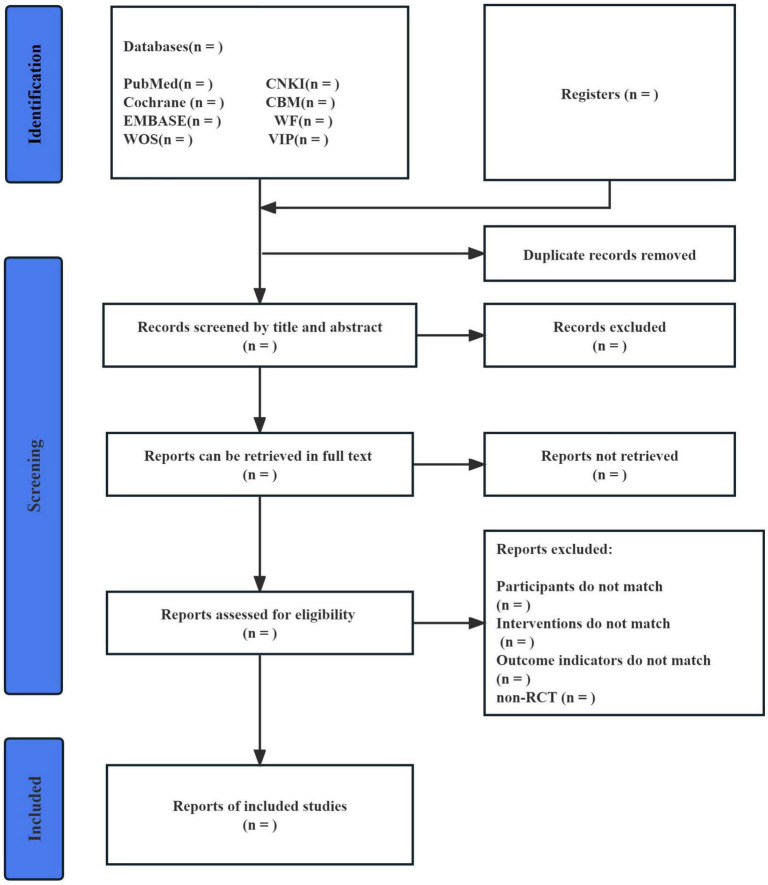
Flow diagram of study selection.

Two reviewers (WC and XD) will work independently on literature screening using Endnote software (V.X9.0). After reading the title and abstract of the literature, studies that do not meet the inclusion criteria will be removed. For studies that may meet the inclusion criteria, further review the full text to decide whether to include. In case of disagreement, a third reviewer (XG) will read and discuss it before making a decision. The process of study selection will be shown in a PRISMA diagram (Figure 1).

### 2.5 Data extraction and management

Data extraction and management will be completed by two reviewers (WC and XD) independently. If there is any inconsistency, two reviewers will check the original text at the same time and ask a third party arbitrator (XG) to discuss and solve it. Data will be extracted in a special data extraction table, including author name, year of publication, randomized method, diagnostic criteria, sample size, age of subjects, intervention measures, duration of treatment, and outcome indicators.

### 2.6 Quality assessment

The risk of bias will be assessed using the approach recommended by *Cochrane Handbook for Systematic Reviews of Interventions* (33). It will mainly include seven items: random sequence generation (selectivity bias), allocation concealment (selectivity bias), blinding of participants, carers or people delivering the interventions (implementation bias), blinding of outcome assessors (measurement bias), missing outcome data (attrition bias), selection of the reported result (reporting bias), and other sources of bias (other bias). Evaluate each case as “low risk,” “unclear risk,” or “high risk.” In the event of disagreement, the third reviewer will review and discuss it before reaching a decision.

### 2.7 Dealing with missing data

When missing data is available, we will make an effort to contact the original author of the study via email to obtain the necessary information. If not available, we will use sensitivity analysis to assess the impact on overall treatment efficacy of including trials with unreported intent to analyze treatment, high participant turnover, or other missing data.

### 2.8 Assessment of heterogeneity

We will assess the heterogeneity among the included studies. If *P* > 0.1 and *I*^2^ ≤ 50%, we will employ a fixed-effect model. If *P* ≤ 0.1 and *I*^2^ > 50%, indicating substantial heterogeneity among all groups, we will conduct subgroup analysis or sensitivity analysis to identify the source of heterogeneity. If heterogeneity is still high, a random-effect model will be chosen.

### 2.9 Data synthesis

For statistical analysis of each outcome, we will utilize RevMan 5.4, a statistical software provided by the Cochrane Collaboration. Continuous variables will be expressed as mean difference (MD), dichotomous variables as relative risk (RR), and 95% confidence interval (CI) will be used for each effect size. Statistical significance will be considered at *P* < 0.05.

### 2.10 Subgroup analysis and sensitivity analysis

We will use subgroup analysis to explore possible sources of heterogeneity, based on the following. (1) Patient characteristic (age, education level, severity of incontinence). (2) Types of acupuncture (Acupuncture, Electroacupuncture, Moxibustion, Body Acupuncture, Abdominal Acupuncture, Warm Acupuncture, Fire Needling, etc.). (3) Types of control group (Kegel pelvic floor muscle training, biofeedback training, low-frequency bioelectrical stimulation, vaginal dumbbells). (4) Duration (30 days vs. < 30 days).

If substantial heterogeneity persists following subgroup analysis, we will conduct a sensitivity analysis to evaluate the robustness of the pooled results. Studies deemed to have a high risk of bias, as well as studies with missing data, will be systematically excluded.

### 2.11 Assessment of publication bias

If the results of the meta-analysis include more than 10 studies, publication bias analysis will be performed using the funnel plot. *P* < 0.05 will be considered statistically significant.

### 2.12 Grading the quality of evidence

We will assess the quality of evidence using the *Grading of Recommendations Assessment, Development, and Evaluation methodology (GRADE)*. All results will be divided into four levels: high, moderate, low and very low.

## 3 Discussion

With the implementation of China’s three-child policy in 2021, experts predict there may be a brief spike in the number of births. This may put some pressure on OB/GYNs in China (34). Therefore, the occurrence of PSUI has attracted much attention. Although PSUI is not life-threatening, it can seriously affect patients’ physical and mental health and quality of life (35). Traditional Chinese medicine believes that the occurrence of this disease is mainly caused by postpartum Qi and blood deficiency, kidney injury over time and damage bladder; gasification function. *“Zhu Bing Yuan Hou Lun”* said, “Because giving birth used a lot of Qi (energy), that may hurt the bladder function, and may lead cold air into the uterus system caused the vesical sac and bladder loses function, so enuresis And or manty are caused by difficulties in childbirth.” This disease should be treated with tonifying qi and tonifying kidney, receiving astringent and tonifying spleen and stomach. Acupuncture treatment of PSUI has the effects of tonifying deficiency and draining excess, regulating qi and blood, and balancing Zang-Fu Yin and Yang. New studies believes that acupuncture may promote the repair of pelvic tissue and bladder function by reducing serum relaxant and pelvic tissue transforming growth factor (TGF-β1) levels (36). Electroacupuncture stimulation can alleviate the signs of SUI, and its mechanism is related to the degradation of collagen in the anterior vaginal wall (37). Another study found that EA treatment changed the species composition of the intestinal flora in rats with SUI. Whether there is a link between the influence of EA on intestinal flora and the regulation of collagen metabolism is still unknown (38). The pelvic floor rehabilitation training can improve the pelvic floor blood circulation and muscle excitability by increasing the tension and contractility of the pelvic floor muscles, so as to relieve the symptoms of urinary incontinence. There was a study revealed that tremendous potential for the improvement of PFME education and targeting at-risk women in the peripartum period (39). Studies have shown that the combined treatment of PSUI can achieve mutual consolidation and prolong the curative effect. The significance of this systematic review is to provide evidence for clinicians to choose acupuncture combined with pelvic floor rehabilitation training to improve post-partum stress urinary incontinence.

Advantages of this study include: (1) This review will provide a comprehensive assessment of the efficacy and safety of acupuncture combined with pelvic floor rehabilitation training in the treatment of PSUI. (2) The search strategy of this review is comprehensive, and 8 databases and clinical trial registration platforms will be searched. (3) Outcome indicators consistent with clinical practice will be used in this study, including ICI Q-SF, 1-h urine pad test and pelvic floor muscle potential value.

There are still limitations to this study. (1) We will only search the Chinese and English databases, which may result in language sampling bias. (2) Due to the particularity of acupuncture operation, this review mainly considers the types of acupuncture, the experience and skills of acupuncture operators, and the specific acupuncture points are not limited. (3) Due to the limited literature available, the mechanism of PSUI cannot be discussed in depth in this review.

In future studies, the database of other languages should be increased to reduce language sampling bias. With the continuous research on PSUI, we should further explore the mechanism of its occurrence and the mechanism of acupuncture combined with pelvic floor rehabilitation training in the treatment of PSUI.

## Ethics statement

Since we do not collect primary data for this systematic review, ethical approval is not required. The results of this protocol will be published in a peer-reviewed journal in accordance with PRISMA guidelines.

## Author contributions

CW: Writing−original draft. DX: Writing−original draft. GL: Methodology, Writing−review and editing. LY: Formal analysis, Writing−review and editing. GX: Supervision, Writing−review and editing.
